# MASLD: insights on the role of folate in hepatic lipid metabolism

**DOI:** 10.3389/fnut.2025.1583674

**Published:** 2025-07-16

**Authors:** Minlan Yang, Xiaoyu Xiao, Jie Mei, Pubu-ciren, Quan Gong

**Affiliations:** ^1^School of Medicine, Yangtze University, Jingzhou, China; ^2^Shannan Maternal and Child Health Hospital, Shannan, China

**Keywords:** MASLD, folate, hepatic lipid metabolism, one-carbon metabolism, methionine metabolism

## Abstract

Metabolic dysfunction-associated steatotic liver disease (MASLD) is also known as fatty liver disease associated with non-alcoholic fatty liver disease (NAFLD), which is a spectrum of chronic liver diseases characterized by steatosis, inflammation, fibrosis and liver injury. The incidence and prevalence of MASLD is increasing rapidly worldwide. It is a multifactorial disease and there is no single drug approved for its treatment. The liver is the main organ that stores and metabolizes the B9 vitamin folate, which is synthesized mainly from dietary nutrients and intestinal microbiota and plays an important role in processes such as nucleic acid synthesis, methylation, and one-carbon metabolism (OCM). Serum folate levels are generally low in MASLD patients, and the low levels of endogenous folate lead to abnormalities in methionine metabolism and OCM, which disrupt lipid metabolism signaling pathways, and cause abnormalities in hepatic lipid metabolism, which may be related to the occurrence of metabolic disorders such as MASLD. Target folate may have beneficial effects in regulating hepatic lipid metabolism through regulating methionine metabolism, OCM and DNA methylation, and signaling pathways. Though a handful of studies argue that folate supplementation had no effect on blood pressure and lipids in patients with metabolic diseases, majority suggest that folate has the potential to serve as a potential therapeutic agent for the development of MASLD and the onset of metabolic associated steatohepatitis (MASH). To date, further research is needed in MASLD to (a) establish the dose of folate as a treatment, (b) determine the duration of therapy, especially in individuals with metabolic diseases, and (c) test its benefit on the different component features of MASLD (hepatic fat, inflammation, and fibrosis).

## 1 Introduction

The liver is an important organ involved in the regulation of lipid metabolism, which can convert carbohydrates such as glucose into triglycerides (TGs) and cholesterol, and then secrete it into the blood for peripheral tissue storage and utilization. Dysregulation of hepatic lipid metabolism leads to metabolic diseases such as fatty liver, hyperlipidemia and diabetes. Metabolic dysfunction-associated steatotic liver disease (MASLD) is also known as fatty liver disease associated with non-alcoholic fatty liver disease (NAFLD). With the global epidemics of obesity and type 2 diabetes mellitus (T2DM), the prevalence of metabolic disorders and fatty liver disease in patients with other types of liver disease is increasing. The limitations of “NAFLD” are becoming increasingly evident, seriously affecting the diagnosis, and prevention of fatty liver disease. In addition, the term “alcoholic” in the NAFLD is stigmatized in the Western. The “A multi-society Delphi consensus statement on new fatty liver disease nomenclature” was released at the EASL Annual Meeting in 2023, and the new consensus recommends that NAFLD be renamed MASLD (1). A large number of studies have concluded that the serum folate level is generally decreased in patients with MASLD, and a higher serum folate level is negatively correlated with MASLD (2). Therefore, a better understanding of the mechanism of folate in regulating lipid metabolism during the development of MASLD may help identify new treatment strategies. In our previous published review, we had summarized the role of folate in various liver diseases through different mechanisms, but less mention has been made of the mechanisms of folate regulating hepatic lipid metabolism (3). In this review, we review the roles of folate on hepatic lipid metabolism, and the potential efficacy of folate supplementation in the treatment of MASLD. We focus on the direct effects of folate on hepatic lipid metabolism, and suggest that targeting folate may have a significant effect on alleviating MASLD.

## 2 Folate in physiology

Folate, chemically known as pteroylglutamate, is a water-soluble B-family vitamin. As a group of methyl transmitters in one-carbon metabolism (OCM), folate is involved in purine and pyrimidine synthesis, as well as DNA methylation. Natural folate is found in various reduced forms in vegetables, fruits and other foods, it is unstable and easily degraded when exposed to acid, heat or light. Synthetic folate, known as folic acid, is found mainly in pharmaceuticals and some fortified foods. Compared with natural folate, it has the advantages of stability and high absorption. Folate intake is mainly metabolized and converted in the liver. After ingested dietary natural folate enters the intestine, polyglutamic acid needs to be hydrolyzed to monoglutamic acid, and then absorbed by active transport through the intestinal mucosa (4). Before entering the blood, the monoglutamate form is reduced to 5-methyltetrahydrofolate (5-MTHF) in the liver, which is mediated by methionine synthase (MTR), which transfers a methyl group to homocysteine (Hcy) to produce tetrahydrofolate (THF) and methionine. THF is the active form of folate. It is an important intermediate involved in OCM, and can be used in the synthesis of purine and thymidylate. 5-MTHF also participates in the synthesis process from deoxyuridine monophosphate (dUMP) to thymidylic acid (dTMP). The deficiency of 5-MTHF leads to the obstruction of dTMP synthesis (5). Therefore, folate are essential for the synthesis and repair of DNA damage and for the subsequent methylation of DNA and other molecules.

The methionine produced by folate metabolism in the liver is further converted to S-adenosylmethionine (SAMe). As a methyl donor of SAM-dependent methyltransferases (MTases), SAMe transfers methyl groups to nucleophilic receptors and initiates the methylation process. Methylation is one of the most important chemical reactions, which is involved in almost all life processes, and most of the methylation reactions are made of SAMe as the methyl source. After methyl transfer, SAMe is transformed into S-adenosyl-l-homocysteine (SAH), which is then hydrolyzed to Hcy and adenosine.

Vitamins play a crucial role in lipid metabolism, which are involved in the synthesis and oxidation of fatty acids and regulate lipid metabolism through various mechanisms. Vitamin C promotes the conversion of cholesterol into bile acid by participating in the microsomal respiratory chain (6). Vitamin D regulates adipose differentiation, adipokine secretion, and inflammation in adipose tissue (7). B-family vitamins provide methyl radicals necessary for balanced phospholipid biosynthesis through conversion to coenzymes such as vitamin B12 and folate through methionine synthesis. Obese people are generally deficient in micronutrients, and their serum level and intake of folate are low.

## 3 Folate in hepatic lipid metabolism

### 3.1 Hepatic lipid metabolism

Hepatic lipid metabolism mainly includes *de novo* synthesis of fatty acids using acetyl-CoA as raw materials, fatty acid uptake, very low density lipoprotein (VLDL) secretion, and fatty acid *β* oxidation (8). Fatty acids are mainly synthesized from carbohydrates in fat tissue, liver and lactating mammary glands. Carbohydrates are converted to acetyl-CoA through glycolysis. Acetyl-CoA is then generated by the tricarboxylic acid (TCA) cycle to produce citric acid, which is subsequently converted back into acetyl-CoA by ATP-citrate lyase in the cytoplasm. This process further elongates the fatty acid chain by two carbons. Under the catalysis of fatty acid synthase, malonyl-CoA and one acetyl-CoA are condensed to form palmitic acid. Palmitic acid undergoes C-chain extension and desaturation through essential fatty acid desaturases, such as stearoyl-CoA desaturase-1 (SCD1) and fatty acid desaturases (FADs), to generate various other fatty acids. Fatty acids are transported to hepatocytes primarily by membrane-binding transporters. Fatty acid uptake is mediated by various transporters such as the fatty acid receptor CD36, fatty acid transport proteins (FATPs), and fatty acid-binding proteins (FABPs). FABPs allow fatty acids to remain soluble and be transported to individual organelles. FATPs are transporters responsible for transporting long-chain fatty acids. Abnormally elevated transporters increase fatty acid uptake and lipid accumulation in hepatocytes, promoting the occurrence and development of MASLD.

Fatty acid oxidation is the primary process of hepatic lipid metabolism, by which fatty acids break down into CO_2_ and H_2_O, releasing large amounts of ATP. Fatty acids are catalyzed to form fatty acyl-CoA by fatty acyl -CoA synthase, which is located in the endoplasmic reticulum and the outer membrane of mitochondria. After entering the mitochondrial matrix, the fatty acyl-CoA is catalyzed by the fatty acid *β*-oxidation system to from β-ketoacyl-CoA, then being decomposed to produce acetyl-CoA and fatty acyl-CoA. Acetyl-CoA, the product of fatty acid oxidation, enters the TCA cycle and undergoes complete oxidation. FATPs are regulated by nuclear receptor peroxisome proliferation-activated receptor-*α* (PPARα). When deficient in nutrients, PPARα in the cytoplasm translocates into the nucleus and upregulates the expression of genes related to fatty acid oxidation, thus facilitating fatty acid oxidation to uphold the homeostasis of hepatic lipid metabolism (9).

The MASLD is characterized by the excess accumulation of fat in hepatocytes and may progress to metabolic dysfunction-associated steatohepatitis (MASH). If the accumulated lipids in the liver cannot be metabolized and cleared in time, the accumulation of lipids will lead to hepatic steatosis, which is an important component of MASLD and MASH, is caused by an imbalance between intrahepatic TGs production and secretion. Altered lipid profiles in the progression of MASLD to MASH. An increase in the levels of molecular species of TGs containing shorter-chain saturated and monounsaturated fatty acids was observed in patients with MASLD. Nevertheless, decreased levels of TGs containing polyunsaturated fatty acids (PUFAs), including *ω*-3 and ω-6 fatty acids were found in patients with MASH. However, the ω-6 to ω-3 ratio in TGs was elevated in patients with MASH. Given the relevance of TGs content to the development of MASLD and MASH, these lipids may be used as biomarkers for the non-invasive diagnosis of MASLD and MASH (10). The various steps of hepatic lipid metabolism are regulated by the interaction of hormones, nuclear receptors, intracellular signaling pathways, and transcription factors.

### 3.2 Low levels of folate in MASLD

MASLD exhibits pathological characteristics related to fat degeneration and storage. According to the progression, MASLD mainly consists of three stages: non-alcoholic fatty liver (NAFL), MASH, and hepatic fibrosis. MASLD is the result of a combination of genetic, environmental, and dietary factors. Its pathogenesis and development mainly involve insulin resistance (IR), abnormal lipid metabolism, oxidative stress, inflammation, and disorders in intestinal flora.

Clinically, patients with MASLD are often associated with obesity, diabetes, or metabolic syndrome. Obese and overweight patients have significantly lower serum folate levels (11). One study including 146 adult participants aged 20 years and older in the National Health and Nutrition Examination Survey (NHANES) from 2011 to 2018 concluded that serum folate and 5-MTHF levels were also negatively correlated with MASLD (2). Depending on the further progression, MASH can advance rapidly, leading patients to develop hepatic fibrosis and cirrhosis. Advanced hepatic fibrosis (AHF) is closely associated with the prognosis and mortality of patients with MASLD. A cross-sectional study of 5,417 participants aged 18 years and older based on 2011–2018 NHANES data showed that people with high serum folate levels had a reduced risk of developing MASLD and AHF (12), whereas in this report, abnormally high serum folate levels (> 50 ng/mL) was excluded. A cross-sectional analysis of 6,610 participants aged 18 years and older in the NHANES database from 2011 to 2018 found that serum total folate and 5-MTHF were negatively correlated with the prevalence of MASLD, while a higher unmetabolized folate (UMFA) concentration was significantly correlated with a higher prevalence of MASLD (13). Analyzing 549 participants in the 2017–2018 NHANES database, it showed that BMI significantly mediates the relationship between 5-MTHF and hepatic fibrosis. Serum total folate or 5-MTHF is negatively associated with hepatic steatosis or fibrosis in adolescents (14).

Animal experiments have verified the above conclusions. Increased liver lipid accumulation and decreased liver and serum folate levels were also found in a high-fat diet (HFD)-induced obesity mouse model (15). Folate deficiency decreased the activity of glutathione peroxidase and increased lipid peroxidation in the liver. The increase of plasma Hcy and the decrease of plasma and liver folate levels in folate-deficiency rats were significantly correlated with the increase of lipid peroxidation in the liver (16).

Reduced serum folate levels have been observed in patients with MASLD and animal models, indicating a possible relationship between disruptions in folate status and the progression of MASLD. Therefore, folate supplementation may be expected to reduce liver injury and slow the progression of MASLD.

### 3.3 Gut microbiota-folate-liver axis regulates lipid metabolism

In addition to dietary nutrients, the synthesis of B-family vitamins, including folate, by gut microbiota has been recognized. As a result, germ-free animals lacking microbiota require supplements of vitamin K and certain B vitamins that their traditional counterparts with intact microbiota do not need. The genera of bacteria commonly found in the distal intestine, including Bacteroides, Bifidobacterium, and Enterococcus, can synthesize vitamins (17). An in-depth analysis of vitamins produced by human gut microbes highlights the importance of evaluating the folate synthesis capacity of various gut cells. Many common human gut bacteria have the ability to synthesize B vitamins, and the metabolism of B vitamins by intestinal flora also varies with age. The infant intestinal flora is rich in genes for *de novo* synthesis of folate, while the adult flora is rich in genes related to the metabolism of folate and its reduced form THF (18).

A large number of animal experiments have been reported, changes in intestinal flora may affect lipid metabolism, which could play a crucial role in the onset and progression of MASLD. Changes in microbiota composition through the use of prebiotics, such as inulin-type fructans, can decrease steatosis and lipogenesis (19). In rats fed with a HFD, supplementation with the prebiotic fructooligosaccharide (FOS) reduced body weight and fat content, and increased CD36 expression in the small intestine. Both FOS altered the small intestine microbiota and increased the relative abundance of Bifidobacterium. *Bifidobacterium pseudolongum* can enhance small intestinal nutrient sensing and regulate food intake to improve energy homeostasis (20). The levels of TGs and VLDL in the plasma of rats fed with prebiotics decreased. Prebiotics, as probiotic stimulants, have the function of promoting the proliferation of probiotics and improving intestinal flora. Specific probiotics such as Lactiplantibacillus, Lactococcus and Bifidobacterium are highly efficient in synthesizing folate. These microbial folate, due to their natural structure, are more readily absorbed into the intestines than chemically synthesized folic acid and avoid the risk of toxicity associated with the accumulation of unmetabolized folic acid. Prebiotics also inhibited enzymes associated with lipogenesis, such as acetyl-CoA carboxylase (ACC) and FAS (21), which led to the inhibition of fatty acid synthesis and consequently reduced TGs content.

Clinical trials also demonstrated that prebiotic feeding for 8 weeks significantly reduced liver inflammation, decrease of hepatic enzymes and steatosis and fibrosis (22). Supplementation with Bifidobacterium and prebiotics to regulate the gut microbiota significantly reduced the levels of inflammatory factors, steatosis, and MASH (23). Therefore, target the metabolic activity of microorganisms may help regulate liver fat production and potentially hinder the development of steatosis.

**Figure 1:**
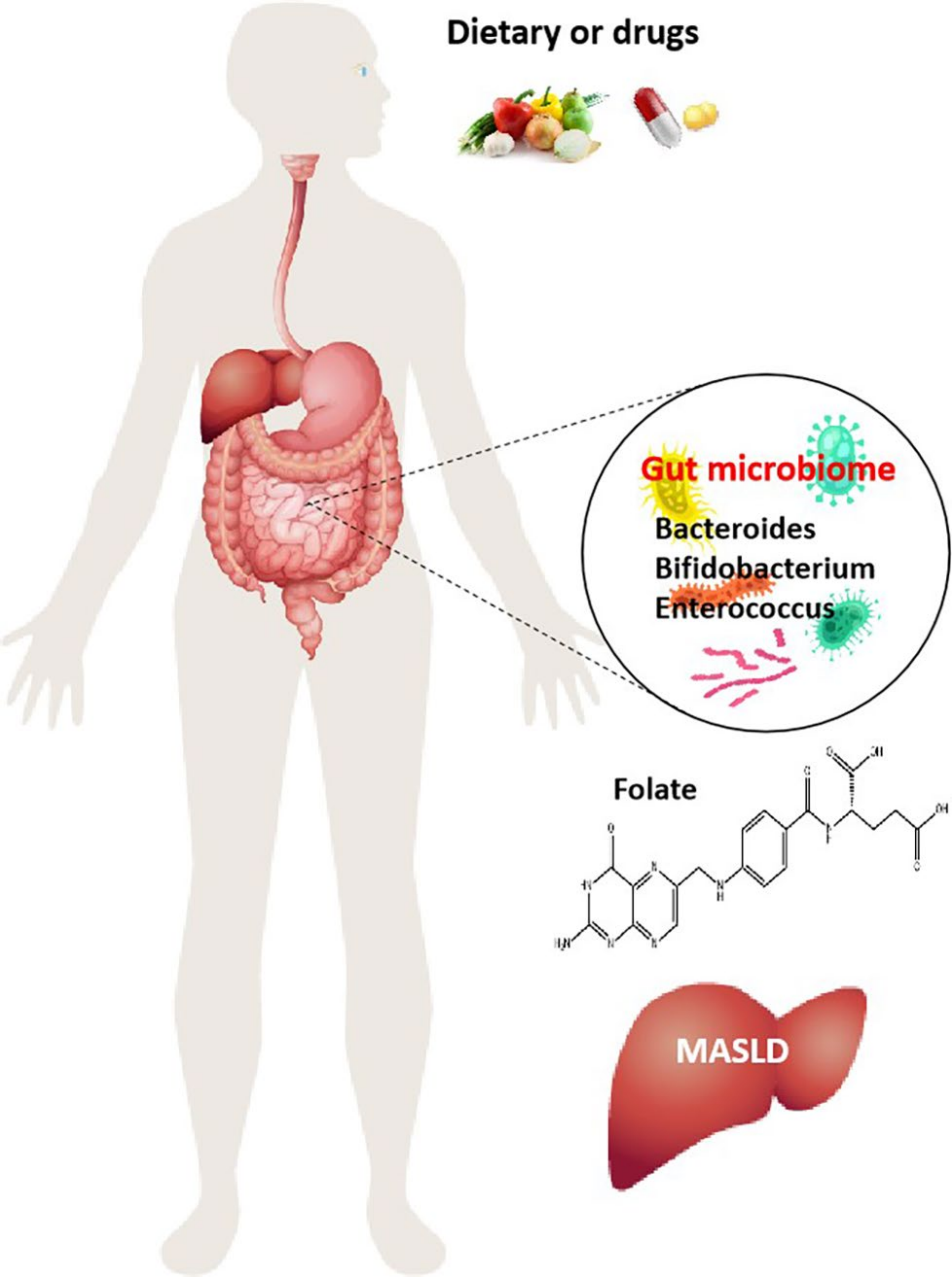
Role of folate produced by gut microbiota in MASLD. Dietary and drugs induce alterations of the intestinal microenvironment and changes in the composition of gut microbiome, increase the number of folate-producing organisms, Bacteroides, Bifidobacterium, and Enterococcus, and then folate is involved in the regulation of MASLD onset and progression.

The progression of MASLD is also accompanied by changes in intestinal microbes, and intestinal microbial transplantation can inhibit the progression of MASLD. In the MASLD mice induced by a HFD, it was found that there is a positive correlation between the abundance of *Bacteroides thetaiotaomicron* and the remission of the metabolic syndrome. *B. thetaiotaomicron* regulates the intestinal microbial composition and induces a decrease in the Firmicutes/Bacteroidetes ratio in the gut of mice with MASLD. Bacteroides thetaiotaomicron reduces body weight and fat accumulation, lowers hyperlipidemia and insulin resistance, and prevents hepatic steatohepatitis and liver injury. In addition, *B. thetaiotaomicron* can also enhance enterohepatic folate and unsaturated fatty acid metabolism to improve MASLD (24). *Ganoderma lucidum* heteropterpene inhibits *de novo* lipogenesis in the liver, promotes fatty acid oxidation and low-density lipoprotein transport, and has a significant effect on inhibiting hepatic steatosis. Oral ganoderma meroterpene derivatives (GMD) improved MASLD by reducing endotoxemia, enhancing lipid oxidation, and decreasing lipid synthesis and output in the liver. The enrichment of GMD bacteria such as *B. thetaiotaomicron*, *Bacteroides dorei*, and *Bacteroides uniformis* is the primary factor contributing to the increased intestinal folate levels. At the same time, the folate-mediated OCM was also significantly enhanced (25). These results suggest that folate synthesis by intestinal bacteria mediates OCM and regulates hepatic lipid metabolism, which is also one of the main mechanisms of folate in slowing down the progression of MASLD. In addition, the low-carb diet, which limits carbohydrates, also promoted a significant increase in the abundance of folate-producing Streptococcus in the gut microbes of patients. The increase in folate concentration would lead to changes in hepatic metabolism, inhibiting lipid synthesis, and accelerating the oxidative decomposition of lipids. Some studies have found that an increase in serum folate accounts for 35.4% of the reduction in liver fat content. A low-carb diet can quickly shift intestinal microbes toward folate production, significantly inhibits fatty acid synthesis, and promotes fatty acid oxidation (26). Thus, regulating folate and folate metabolism by interfering with gut microbes such as Bacteroides, Bifidobacterium, and Enterococcus may help ameliorate MASLD (Figure 1).

### 3.4 Folate metabolism regulates lipid metabolism

#### 3.4.1 Folate regulates lipid metabolism

**Figure 2:**
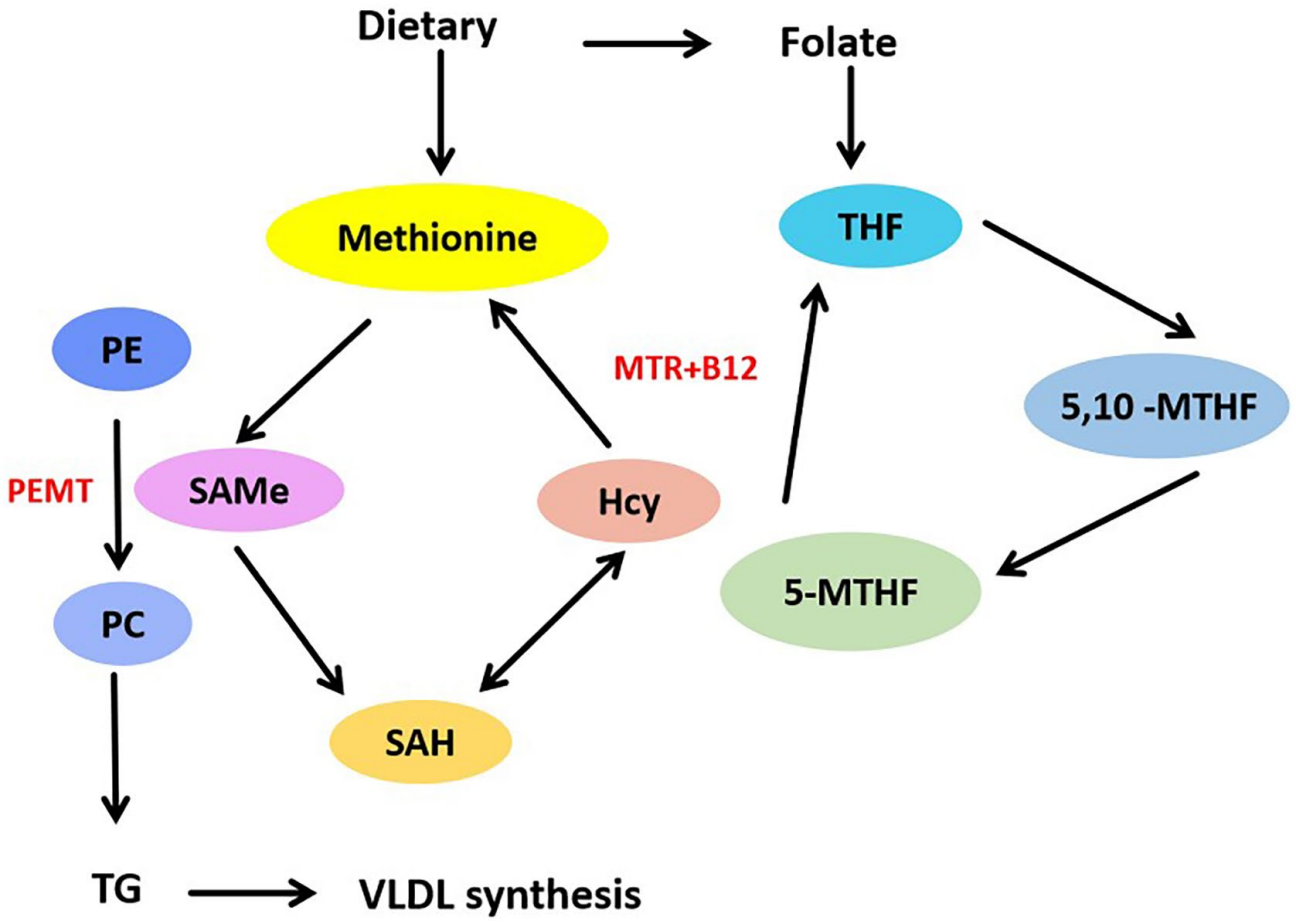
Folate mediates methionine metabolism to promote VLDL synthesis. Methionine from dietary can be converted into SAMe, SAMe is catalyzed by methyltransferase, SAMe demethylates to produce SAH, SAH deadenosine to produce Hcy. Hcy accepts methyl group to reproduce methionine, this is methionine cycle. Through the folate cycle, MTR catalyzed the transfer of methyl group from 5-MTHF to Hcy, and change to THF. PEMT used SAMe to catalyze the methylation of PE into PC, thus promoting the synthesis of VLDL.

Low serum folate levels are often associated with obesity. Increased liver lipid accumulation and decreased liver and serum folate levels were found in HFD-induced obese mice. The obstruction of folate transport results in reduced folate storage in the liver (19). Lack of folate can induce increased secretion of pro-inflammatory factors and impair hepatic lipid metabolism, resulting in hepatic lipid accumulation and fibrosis. Rats fed a folate-deficient diet exhibited fatty infiltration, increased TGs, and decreased phospholipid methylation of the liver. It suggests that folate deficiency blocks phospholipid synthesis in the liver. Folate consumption is associated with high expression of genes involved in lipid biosynthesis. Dietary folate deficiency leads to changes in hepatic fatty acid metabolism, DNA synthesis, and circadian rhythm (27). In addition, VLDL transport was impaired in folate-deficient mice (28), suggesting that folate may be involved in regulating VLDL synthesis and transport. Folate is essential for the synthesis of SAMe from methionine. Phosphatidyl ethanolamine N-methyltransferase (PEMT) utilizes SAMe as a methyl donor to catalyze the methylation of phosphatidyl ethanolamine (PE) to phosphatidylcholine (PC) (29), a process essential for VLDL formation in hepatocytes. As showed in Figure 2. The process of PC synthesis is impaired in folate deficiency, leading to a decrease in the PC/PE ratio. This imbalance may impact the lipid production of VLDL and contribute to hepatic lipid accumulation (30). Loss of folate transporter solute carrier family 19 member 1 (SLC19A1) in hepatocytes reduces intracellular folate levels, impairs lipid metabolism, and leads to the accumulation of lipid droplets in hepatocytes (31). The mitochondrial folate enzyme aldehyde dehydrogenase 1 family member L2 (ALDH1L2) converts 10-formyltetrahydrofolate (10-formyl-THF) to THF and carbon dioxide. Mitochondrial overexpression of ALDH1L2 produces sufficient NADPH to maintain high levels of glutathione, which is essential for supporting high levels of cysteine, the precursor of coenzyme A. Abnormalities in hepatic lipid metabolism were found in ALDH1L2 knockout mice. Knockout of ALDH1L2 led to dysregulated lipid metabolism and reduced ATP levels in the mitochondria (32). This finding also confirms that folate metabolism regulates lipid metabolism and energy homeostasis in the liver.

Folate deficiency also affects fetal development. The folate and methionine cycles are activated in the maternal liver, and low folate levels may alter maternal hepatic metabolism. SREBP1 and ACC1 expression is reduced in the livers of pregnant rats fed a folate-deficient diet. Folate deficiency inhibits lipid synthesis, leads to disturbed protein regulation and abnormal gene expression in the liver, which elevates TGs levels and lowers plasma HDL, resulting in steatosis (33). Besides, folate deficiency increases lipid accumulation and leptin production in fat cells (34). Thus, changes in hepatic lipid metabolism may indirectly affect fetal development. The dietary with high folate and low vitamin B12 reduced total fatty acids and desaturase activity in the maternal liver, but had the opposite effect on the offspring. An unbalanced prenatal and postnatal folate/vitamin B12 diet plays a crucial role in regulating hepatic desaturase gene expression and enzyme activity associated with lipid metabolism in the adult offspring (35).

#### 3.4.2 Methionine metabolism regulates lipid metabolism

Methionine is an essential amino acid. In addition to serving as a substrate for protein synthesis, methionine is a primary methyl donor. The folate cycle coupled with the methionine cycle involves 5-methyltetrahydrofolate-homocysteine methyltransferase (MTR), which catalyzes the transfer of a methyl group from 5-MTHF to Hcy. Methionine can be further activated and converted into SAMe, a crucial substrate that regulates methylation. Lipid metabolism is the main biological process affected by methionine restriction (MR) (36). In adipose tissue, MR increases adipogenesis and fatty acid oxidation. The expression of rate-limiting enzymes involved in fatty acid synthesis, such as FAS and ACC1, as well as SCD1, involved in TGs synthesis, are increased. In the preadipose cell line 3 T3-L1, methionine deprivation negatively affects the activity of lipoprotein lipase (LPL) and hormone-sensitive lipase (HSL) (37), which are key enzymes for the hydrolysis of TGs in serum and adipocytes, respectively. Methionine deprivation leads to lipid accumulation in adipose tissue.

But the hepatic lipid metabolism is not quite the same as that of adipocytes. MR decreased plasma leptin and increased adiponectin were found in Fischer 344 rats feeding an-MR diet. Uncoupling protein 1 (UCP1), which is specifically and highly expressed in brown adipose tissue (BAT), was increased in epididymal and inguinal white adipose tissue (WAT) (38). SCD1 is a key enzyme involved in monounsaturated fatty acid synthesis. MR diet reduced SCD1 expression in the liver and decreased the expression of enzymes involved in lipid synthesis. Therefore, MR influences the expression of genes regulating lipid metabolism and promotes the shift from fatty acid synthesis to fatty acid oxidation in the liver. Dietary MR has a protective effect on hepatic steatosis in mice (39). Methionine deficiency increased SAH and Hcy levels, altered the expression of genes involved in OCM and lipid metabolism, leading to lipid accumulation, activated oxidative stress, and endoplasmic reticulum stress responses in the liver (40). This indicates that abnormal methionine levels can lead to lipid accumulation in the liver and contribute to the development of MASLD.

In addition, folate is important for inhibiting MASH and hepatic fibrosis. As people with chronic liver disease are often comorbid with intrahepatic cholestasis (IHC) clinically, and IHC can further exacerbate liver injury. Folate promotes the production of SAMe, and reduced level of SAMe can bring about cholestasis. SAMe reduces the cholesterol/phospholipid ratio of hepatocyte and erythrocyte cell membranes, improve the fluidity of hepatocyte membranes, and also increase glutathione levels, improve the anti-free radical and detoxification ability of hepatocytes. SAMe is conducive to promoting the regeneration of normal hepatocytes and the repair of damaged hepatocytes, and inhibit inflammatory cytokines. Besides, SAMe inhibited human hepatic stellate cells (HSCs) activation and proliferation (41), reduced of carbon tetrachloride-induced liver injury and liver fibrosis (42), and inhibited collagen processing leading to increased ubiquitination and decreased type I collagen secretion (43). This in turn inhibits the onset and progression of liver fibrosis and cirrhosis.

### 3.5 Folate regulates insulin resistance

The liver is an important target organ for insulin. Insulin inhibits glycogenolysis and gluconeogenesis in the liver and promotes glycogen synthesis. When the function of insulin is weakened, it fails to inhibit hepatic glucose output, leading to hepatic IR. Hepatic IR leads to disturbances in glucose and lipid metabolism, and increased lipid synthesis results in hepatic lipid deposition. IR is the pathogenesis of T2DM and the pathological basis of many metabolic-related diseases, such as obesity and MASLD. A cross-sectional study of 1,530 adults enrolled in the NHANES between 2011 and 2012 found an inverse association between serum folate levels and IR in non-diabetic patients (44). Systematic literature search on PubMed, Web of Science, and EMBASE, as well as previous systematic reviews and meta-analyses indicated that folate supplementation significantly decreased insulin and HOMA-IR levels. These results suggest that folate supplementation may benefit glucose homeostasis and reduce IR (45). A folate/vitamin B12 diet was found to significantly decreased insulin, HOMA-IR, and TGs levels (46). However, other studies have reached different conclusions. Folate supplementation was found to have no significant effect on serum liver enzyme levels, hepatic steatosis, IR, and lipids in MASLD patients. A study of olderly Thailand people revealed a significant negative correlation between folate and Hcy levels, serum Hcy levels increased with age. Besides, folate deficiency significantly increases the risk of developing HHcy (47). According to epidemiological studies, the role of folate in the treatment of MASLD is still controversial. Calcium/calmodulin-dependent protein kinase 2 (CAMKK2) is the primary target of calmodulin, which binds to Ca^2+^ and plays a crucial role in calcium signal transduction pathways by modifying various target proteins, such as kinases or phosphatases. CAMKK2 is involved in regulating key metabolic processes such as obesity and glucose homeostasis. Folate deficiency leads to reduced CAMKK2 methylation. However, CAMKK2 methylation is negatively correlated with HOMA-IR index (48). This also suggests that folate can influence the expression of genes related to lipid metabolism and IR by regulating methylation.

### 3.6 One-carbon metabolism (OCM) in lipid metabolism

Disruption of folate metabolism leads to MASLD, including steatosis, steatohepatitis, fibrosis, and cirrhosis. Folate-mediated OCM is also involved in regulating lipid metabolism. A study recruited 421 participants aged 20–40 years in Poznań, Poland, from 2016 to 2018revealed that serum folate was associated with lower total cholesterol (TC), low-density lipoprotein cholesterol (LDLC), TGs, and triglyceride glucose index in overweight/obese individuals, but not in the normal population. The associations between OCM markers, fatty liver index, and lipids differed between normal-weight and overweight populations (49). Low levels of endogenous folate in rodents disrupt OCM, and may be associated with the development of metabolic diseases such as MASLD (50). As methionine, serine, glycine, and choline are the main sources of one-carbon units, methionine and choline deficiency diet model is the most common MASLD model. Drugs such as methotrexate, that disrupt OCM, induce liver injury and fatty liver diseases (51). The one-carbon unit transfer mediated by folate through SHMTs is also essential for mitochondrial biological processes. Reduced mitochondrial content impairs nutrient oxidation and leads to lipid accumulation.

MASLD is associated with dietary folate deficiency and mutations in genes required for OCM. Methionine synthase reductase (MTRR) is a crucial regulator of the methionine and folate cycles, and MTRRgt mutations disrupt folate metabolism and alter overall and locus-specific DNA methylation. MTRRgt mutations previously disrupted OCM, resulting in a wide range of developmental phenotypes and late-onset macrocytic anemia in adulthood. Adult mice with MTRRgt mutation showed decreased glycogen and reduced fatty acid β-oxidation in eosinophilic hepatocytes (52).

Methylenetetrahydrofolate reductase (MTHFR) is a key enzyme in folate metabolism. It catalyzes the reduction of 5,10-MTHF to 5-MTHF and to provide methyl donors for the synthesis and methylation of SAMe. 5-MTHF is the primary form of folate, playing a crucial role in numerous vital biochemical reactions. Thus, there may be a link between altered serum 5-mTHF levels and MASLD progression. Serum5-MTHF was negatively associated with hepatic steatosis or fibrosis (13, 14). All of these indicate that increasing serum 5-mTHF levels may potentially reduce the prevalence of MASLD. Loss of MTHFR enzyme activity is primarily due to genetic mutations. An MTHFR gene mutation leads to low level of folate, and high level of tHcy. Over time, this improper induces higher risk for a variety of diseases. There are many types of mutations in the MTHFR gene, with the most common being the C-T mutation at the 677th nucleotide of exon 4 (C677T). This mutation increases the thermal instability of MTHFR and significantly reduces the enzyme activity of MTHFR. Folate deficiency predisposes men to hepatic fibrosis, which is exacerbated by the MTHFR 677TT mutation. In contrast, the MTHFR 677TT mutation predisposes women to steatosis by altering choline metabolism, leading to abnormal expression of lipid metabolism genes, and promoting hepatocyte steatosis (53). In addition to the mutation in C677T, the mutation in A1298C of MTHFR also affects the MTHFR enzyme activity. When both mutations in C677T and A1298C are present, the effect on the reduced enzyme activity will also be superimposed. MTHFR deficiency results in decreased SAMe, increased SAH, decreased methylation. MTHFR deficiency may exacerbate liver injury through alterations in methylation, inflammatory response, and lipid metabolism. Individuals with MTHFR variants may have an increased risk of liver cirrhosis and its complications (54). In addition, mutation of MTHFR leads to a decrease in the conversion from 5,10-MTHF to 5-MTHF. This reduction lowers the active folate levels and increases tHcy. Therefore, folate supplementation may reduce the elevated serum tHcy levels induced by MTHFR mutation. When excessive folate is supplemented, the level and activity of the MTHFR are inhibited, resulting in accelerated adipogenesis and reduced cholesterol catabolism. Moreover, the effect was more significant in MTHFR-deficient mice (55). In MTHFR-knockout mice, high folate intake hinders the expression of genes involved in cholesterol synthesis and disrupts cholesterol homeostasis in the liver (56). This also suggests that folate deficiency is as harmful as excess, and that excess folate are more harmful to MTHFR-deficient individuals.

### 3.7 Folate regulates signaling pathways in hepatic lipid metabolism

Lipid metabolism includes biological processes involved in lipid synthesis and metabolism of fatty acids, and several different signaling pathways are involved in regulating hepatic lipid metabolism.

#### 3.7.1 AMPK signaling pathway

**Figure 3:**
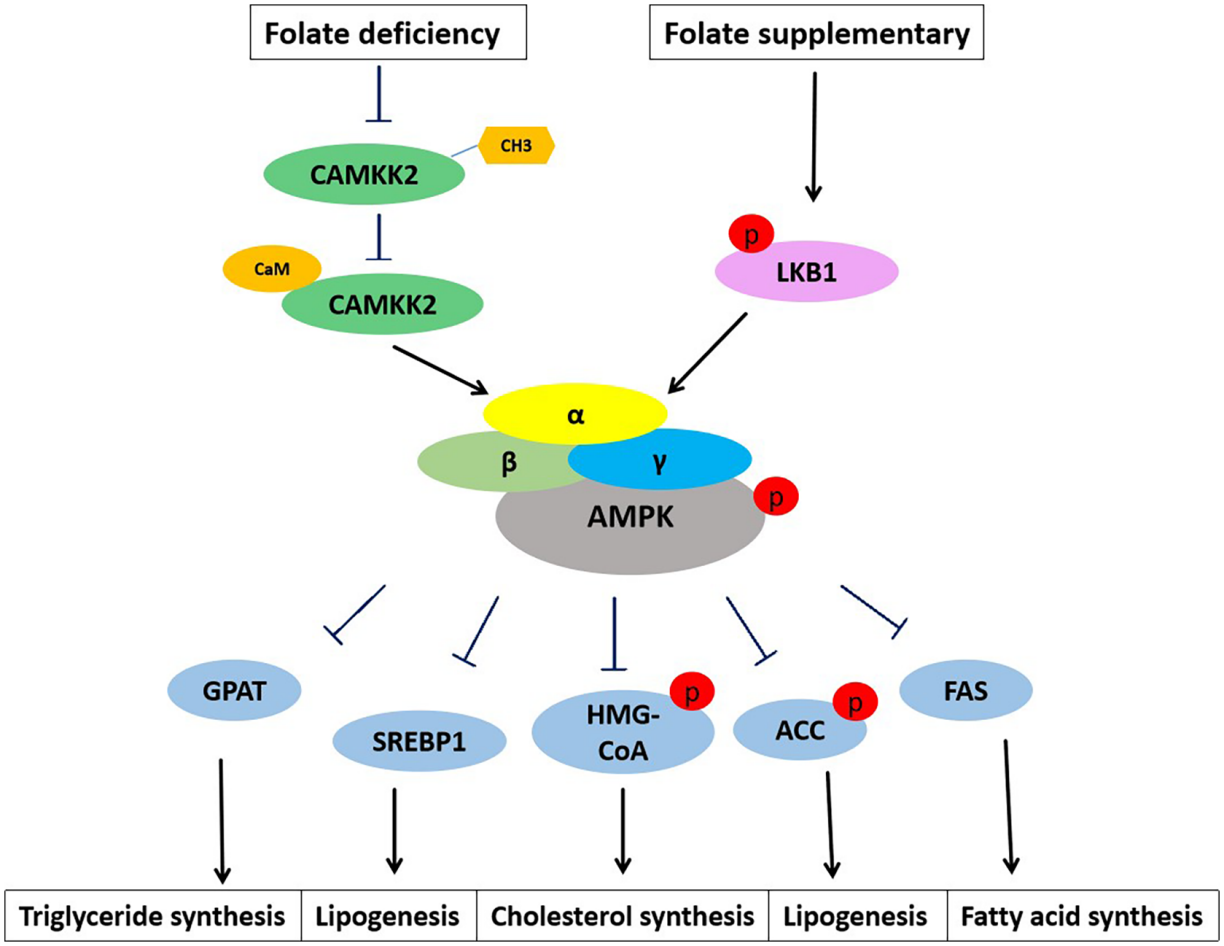
Folate regulates lipid metabolism through AMPK signaling. AMPK appears as a heterotrimer complex containing a catalytic α subunit and regulatory *β* and *γ* subunits. Folate deficiency inhibits CAMKK2 methylation and the binding of CAMKK2 to calmodulin. Phosphorylation of CAMKK2 activates AMPK. The α subunit of AMPK is also easily activated by phosphorylation of upstream kinase LKB1. Activation of AMPK inhibits the expression and activities of lipid metabolism genes, GPAT, SREBP1, HMG-CoA, ACC, and FAS.

Adenosine 5′-monophosphate (AMP)-activated protein kinase (AMPK) is a key regulator of metabolism, associated with energy balance (57). Abnormalities in hepatic lipid metabolism are often associated with dysregulation of AMPK. AMPK inhibits cholesterol and fatty acid synthesis in the liver (Figure 3). AMPK phosphorylates and inhibits the activity of two rate-limiting enzymes: hydroxymethylglutaryl-CoA synthase (HMG-CoA) and ACC. Long-term activation of AMPK also inhibits the expression of cholesterol regulatory SREBP-1, and down-regulates the expression of lipid-related genes such as FAS, pyruvate kinase (PK), HMG-CoA, and ACC. In addition to inhibiting the production of fatty acids, AMPK also inhibits the production of TGs. TGs levels were abnormally elevated, and liver fatty acid production was enhanced when AMPKα2 knockout. AMPK inhibits the transcriptional activity of ChREBP, thereby reducing the conversion of carbohydrates to fat. Meanwhile, AMPK reduced TGs synthesis by inhibiting the glycerol triphosphate acyltransferase (GPAT) of liver mitochondria through phosphorylation (58). These results suggest that AMPK regulates fat deposition in the liver by reducing fatty acid production and promoting fatty acid oxidation. In HFD-induced MASLD, AMPK inactivation is associated with hepatic lipid accumulation, hyperglycemia, and hyperinsulinemia. Folate supplementation restored AMPK activation, thereby improving hyperinsulinemia and lipid and glucose metabolism in HFD-induced mouse (15).

#### 3.7.2 PPARα signaling pathway

MASLD is primarily linked to the downregulation of lipid catabolic pathway genes regulated by PPARα. Methionine metabolism produces SAMe, which is essential for DNA methylation. Methionine supplementation decreased DNA methylation level and promoted the up-regulation of PPARα target genes angiopoietin like 4 (ANGPTL4), fibroblast growth factor 21 (FGF21), and phosphoenolpyruvate carboxykinase 1 (PCK1) in the liver. Methionine supplementation may activate the PPARα signaling pathway through the synthesis of SAMe. The up-regulation of liver PPARα is associated with the improvement of lipid metabolism and immune function (59). These findings suggest that PPARα-regulated metabolic signaling pathways are one of the key mechanisms determining severity of MASLD.

**Figure 4:**
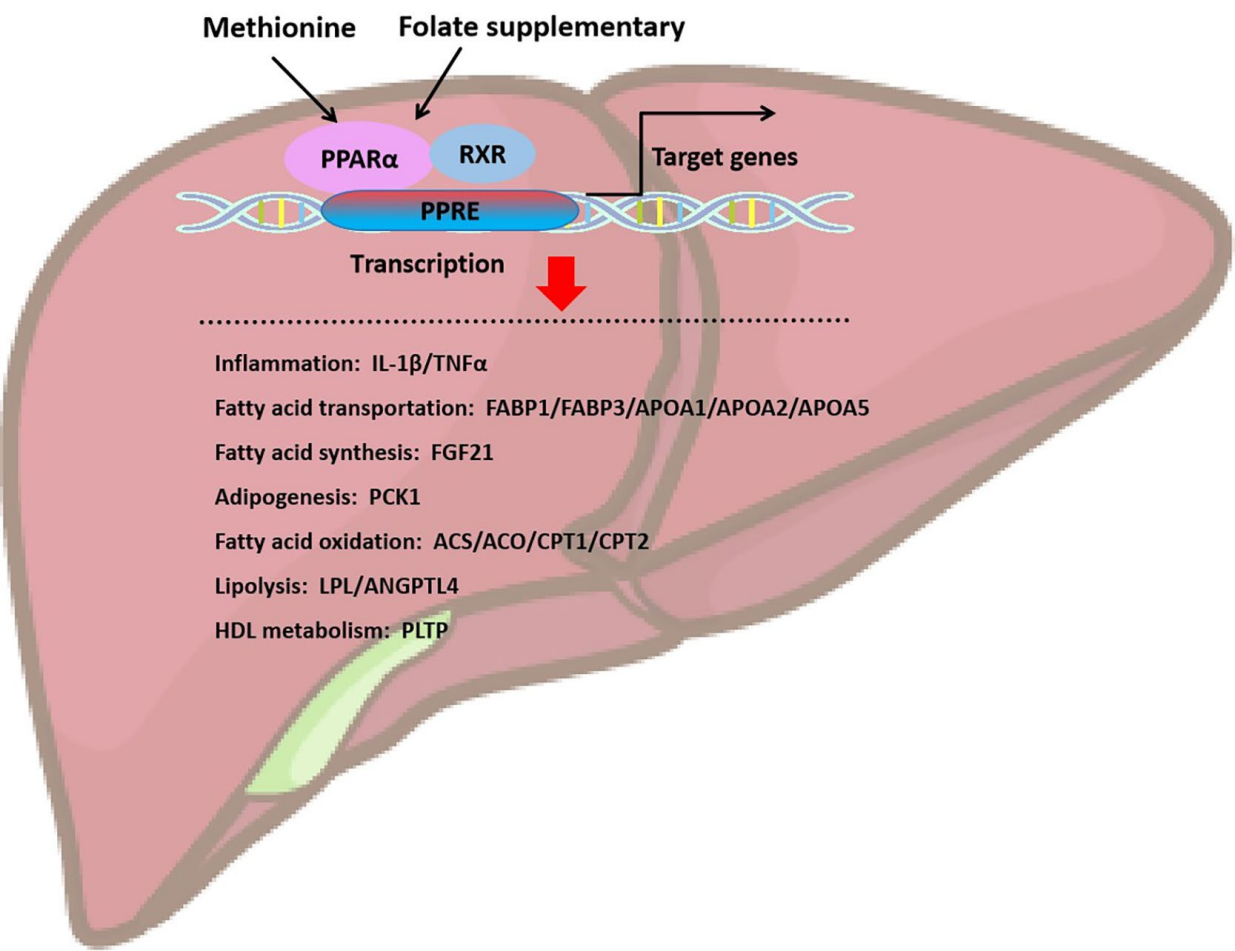
Folate and methionine regulate hepatic lipid homeostasis through the activation of PPARα. Supplementation of folate and methionine activates PPARa, which binds to retinol X-receptor (RXR) to heterodimerize, and PPAR-RXR dimer binds to DNA response element (PPRE) located in promoter or intra gene region to initiate target gene transcription. Target genes are mainly involved in inflammation, fatty acid transportation, fatty acid synthesis, adipogenesis, fatty acid oxidation, lipolysis, and HDL metabolism.

PPARα plays a role in the oxidation and metabolism of fatty acids (Figure 4). Activating PPARα promotes the oxidation of fatty acids and reduces the synthesis of fatty acids, thereby decreasing the level of triacylglycerol. PPARα acts as a transcription factor to stimulate the transcription of target genes. PPARα binds and activates its ligand, then binds to the coactivator to form the PPARα/ligand complex, which enters the nucleus and binds to the PPRE of target gene. The activation of PPRE promotes fatty acid oxidation and reduce fatty acid synthesis (60). PPARα is associated with fatty acid transport, mitochondrial fatty acid oxidative metabolism, inflammatory response, and fibrogenesis. PPARα stimulates fatty acid catabolism by regulating the expression of LPL, apolipoprotein genes (APOA1, APOA2, and APOA5), fatty acid transport and oxidation genes (FABP1, FABP3, ACS, ACO, CPT1, and CPT2), and genes for HDL metabolism (phospholipid transfer protein, PLTP) and ketone synthesis (3-hydroxymethylglutaryl-CoA synthase 2, HMGCS2) (60). Therefore, PPARα may be a key factor in inhibiting liver lipid synthesis and oxidative stress. Kupffer cells, also known as Kupffer-Browicz cells, are macrophages found in the sinusoids of the liver, also play an important role in the development of MASLD. Kupffer cells produce IL1β, inhibit PPAR*α*-dependent fatty acid oxidation, leading to lipid accumulation and TGs synthesis, and promote hepatic steatosis (61). Besides, folate-mediated DNA methylation of PPARα decreased after a folate-restricted diet in pregnant rats and reversed after folate supplementation in the liver. Prenatal nutrition induced differential changes in CpG dinucleotide methylation of the PPARα promoter in the liver of young rats, which persisted into adulthood (62).

#### 3.7.3 Others

Chronic inflammation is an important pathogenic factor in the liver and accelerates the progression of MASLD and MASH, serum levels of many inflammatory markers and mediators, including C-reactive protein (CRP), tumor necrosis factor α (TNFα), IL6 and IL8, Interleukin-1 receptor antagonist (IL1RA) and C-X-C motif chemokine ligand 10 (CXCL10), have been examined as diagnostic markers. As MASLD and MASH is associated with an underlying inflammatory metabolic state, these markers reflect underlying disease pathways, including apoptosis, inflammation, oxidative stress, and abnormal signaling pathways are involved. Serum aminotransaminases (ATs) levels are the most commonly used for assessment of chronic liver diseases, including MASLD and MASH. In a rat model of HFD-induced MASLD, the serum IL22 level decreased, and the autophagy protein LC3B increased in the liver. Folate supplementation significantly reduced the expression of pro-inflammatory cytokines TNFα, C-X-C motif chemokine ligand 8 (CXCL8), and LC3B, and improved hepatitis in a dose-dependent manner (63). A HFD leads to lipid accumulation, activation of the transcription factor nuclear factor kappa-B (NFκB), and increased expression of inflammatory genes in hepatocytes. Supplementing with folate inhibits NFκB activation and significantly reduces the levels of inflammatory cytokines, as well as decreases the accumulation of liver lipids and inflammation (64), and provides antioxidant activity that may help enhance the oxidative decay of mitochondria caused by pro-oxidants (65). This suggests that folate supplementation may regulate the production of pro-inflammatory factors and autophagy, and alleviate the progression of MASLD.

These studies all suggest that folate affects liver function and lipid metabolism by regulating various signaling pathways, thereby influencing the progression of MASLD.

### 3.8 Folate regulates immune response in hepatic lipid metabolism

In addition to hepatocytes, hepatic sinusoidal endothelial cells and HSCs are present in liver tissues and are important cell types involved in the transformation of the hepatic inflammatory response to hepatic fibrosis. The liver is also an important immune organ, containing immune cells such as natural killer cells, macrophages and regulatory T lymphocytes (Tregs), which are involved in the inflammatory response during MASH. Kupffer cells are the most numerous immune cells present intrinsically in the liver and phagocytose dead cell debris and pathogens. Kupffer cells in the initial phase of MASH increase the number of monocytes through the secretion of cytokines, such as TNFα, and monocyte chemotactic proteins (MCPs). Monocyte infiltration of peripheral blood origin, and the use of drugs to target and selectively remove macrophages *in vivo* to attenuate the chronic inflammatory response induced by obesity can reduce liver fibrosis and cirrhosis.

#### 3.8.1 T lymphocytes in MASLD

T lymphocytes are derived from hematopoietic pluripotent stem cells in the bone marrow, are classified as CD4^+^ T and CD8^+^ T cells. In the MASLD mouse model of disordered lipid metabolism, the decline of CD4^+^ T cells in the liver can be related to the exacerbation of their apoptosis by high levels of fatty acids, whereas by inhibiting ROS, it is possible to restore the number of CD4^+^ T cells and to delay the tendency to transform into HCC (66). Thus the absence of CD4^+^ T cells in the hepatic microenvironment may be one of the factors responsible for the important immunomodulatory abnormalities in the progression of MASLD to cirrhosis and HCC. Tregs are a group of immunomodulatory cells, are present in visceral adipose tissue of adult mice. The number of Tregs in the liver of obese mice with MASLD was decreased, suggesting that Tregs influence liver injury during MASLD (66). Th17 cells are a class of T helper cells, secreting a variety of cytokines such as IL17, IL21, and IL22. In MASLD patients, Th17 cells have increased infiltration in the liver and promote localized inflammatory responses by secreting IL17, exacerbating the transition from fatty liver to MASH (67). Other immune cells, including neutrophils, dendritic cells, and natural killer cells have also been found to play an important role in the development and progression of MASLD.

#### 3.8.2 Folate regulates immune cells in MASLD

Although no direct effect of folate on the proportion and number of immune cells in the liver has been reported, a large number of studies have found that folate is involved in the regulation of immune responses. Folate supplementation can increase resistance to infection and enhance immune cell function, indicating that folate is necessary for the proper functioning of the immune system. Folate metabolism is involved in the regulation of antiviral natural immunity, and folate metabolism negatively regulates the antiviral protein 2′-5′ oligoadenylate synthetase (OAS)-mediated antiviral natural immunity by inducing en-dsRNA accumulation (68). In tumor cells, MTHFD2 can efficiently drive the folate cycle and stimulate PDL1 expression to promote tumor immune escape (69). In addition, tumor-associated macrophages (TAMs) express folate receptor β (FRβ), which mediates folate uptake, suggesting that folate conjugates of therapeutic agents are potential immunotherapeutic tools for targeting TAMs (70). Folate deficiency may exert pro-inflammatory signaling by enhancing the monocyte–macrophage system, and the production of the inflammatory mediators, IL1β, IL6, TNFα, and MCP1, and monocytes were significantly increased when folate deficiency (71). Deficiency of folate leads to a decrease in CD8^+^ T cells and their ability to respond to mitogens is inhibited (72), increasing CD4^+^ T/CD8^+^ T cell ratio, which is associated with an increased risk of cancer (73). Although a large number of studies have confirmed that immune cells play an important role in the occurrence and development of liver diseases, there is a lack of reports on the direct regulation of folate on immune cells in MASLD.

#### 3.8.3 OCM regulates immune cells

In addition, studies have reported that the expression of folate transporter proteins varies between macrophage subtypes, with higher levels of 5-MTHF in M2 macrophages, suggesting that folate levels may be associated with the stage of macrophage polarization (74). The OCM enzyme MTHFD2 regulates T cell function, MTHFD2 activates purinergic synthesis and signaling in T cells, promoting inflammatory factor production. In Th17 cells, MTHFD2 inhibits aberrant upregulation of FoxP3. In addition, MTHFD2 deficiency also promotes Treg cell differentiation (75). Blockade of MTHFD2 may curb pro-inflammatory CD4^+^ T cells, while redirecting them toward a regulatory T cell phenotype (76).

Based on the above reported regulatory effects of folate metabolism on immune cells, we hypothesized that folate metabolism may also be involved in regulating the function of immune cells in the liver, which in turn affects liver metabolism. Targeting folate metabolism may delay the onset and progression of liver disease by regulating the number and proportion of immune cells in the liver.

## 4 Target folate on lipid metabolism in MASLD

Nutrition and diet are crucial factors in the prevention and development of MASLD. There are significant differences in the intake of macronutrients and micronutrients between patients with MASLD and HCV.

### 4.1 Folate supplementation regulates lipid metabolism

The progression of MASLD is closely related to the composition of dietary intake (77). Adequate intake of folate may help prevent MASLD (74). Dietary folate and serum folate levels were evaluated in 3,706 adults aged ≥ 20 in the NHANES, serum folate was negatively correlated with TGs and LDLC, and positively correlated with HDLC. Dietary folate was negatively correlated with TC and LDLC (78). Dietary micronutrient supplementation, which generally includes folate, vitamin B6, choline, betaine, and zinc, can reduce weight gain and obesity, improve glucose metabolism, and enhance liver antioxidant capacity and lipid metabolism (79). Supplementation with folate reduced liver adipogenesis and inhibited the proliferation and differentiation of adipocytes (80). Folate supplementation inhibits the synthesis of fatty acids in hepatocytes and coordinates the promotion of hydrolysis and output of TGs to reduce the deposition of TGs. Folate also inhibits the production of fatty acids by weakening the insulin/IGF signal mediated by the PI3K/AKT/SREBP pathway. In addition to folate, inhibitors of IGF2 and PI3K may also prevent the development of MASLD by reducing TGs deposition (81). Oxidative stress in the liver is associated with increased expression of NADPH oxidase. A HFD is associated with weight gain, hepatic lipid peroxidation, liver injury, and significantly increased liver NADPH oxidase activity. Folate supplementation promotes the transcriptional regulation of NADPH oxidase and has a protective effect on liver oxidative stress and liver injury induced by a HFD (82). This also indicates that folate supplementation not only regulates hepatic lipid metabolism by inhibiting lipid synthesis and promoting fatty acid metabolism but also involves a variety of different regulatory mechanisms.

### 4.2 Folate supplementation targets methionine metabolism

Folate is a methyl donor required for the synthesis of methionine, a precursor of SAMe, a substrate for methylation in epigenetic and epigenomic pathways. Methyl donor deficiency can lead to hepatic steatosis and a predisposition to metabolic syndrome. Folate deficiency promotes a decrease in methionine synthase activity, SAMe and SAMe/SAH ratios, hypomethylation of PGC1α, impaired mitochondrial fatty acid oxidation, and induced hepatic steatosis. Hcy is an intermediate metabolite of methionine, and elevated levels of total serum homocysteine (tHcy) regulate cholesterol synthesis in the liver. Folate supplementation reduces Hcy levels, and oral folate supplementation significantly decreases tHcy, serum folate and TC levels in children with hyperhomocysteinemia (HHcy) (83). Folate deficiency increases oxidative stress and Hcy levels. Folate also protects the liver from cholestasis by reducing serum tHcy levels through its antioxidant properties. Folate supplementation inhibits liver fibrosis and improves liver function in rats with cholestasis (84). A high methionine diet induces HHcy, leading to liver injury in rats (85). Liver NADPH oxidase is activated in HHcy, resulting in increased superoxide anion production and peroxynitrite formation. The level of lipid peroxide in the liver of HHcy rats was significantly increased. Folate supplementation can effectively inhibit NADPH oxidase-mediated superoxide anion production, thereby reducing hepatic lipid peroxidation.

### 4.3 Folate supplementation regulates signaling pathways

PPARs are a class of nuclear receptors involved in the physiological processes of lipid metabolism, cell proliferation and differentiation. Folate supplementation may regulate lipid metabolism through PPARs signaling. PPARa regulates the expression of fatty acid oxidation-related genes to promote fatty acid oxidation and maintain hepatic lipid metabolism homeostasis. Folate upregulates PPAR*α* through a SIRT1-dependent mechanism, improves hepatic lipid metabolism, restores hepatic OCM and intestinal flora diversity, and thus alleviates HFD-induced MASH in rats (86). Fenofibrate is a lipid-lowering drug and one of the PPARα agonists, which is a drug target for MASLD. Fenofibrate causes a significant increase in plasma tHcy, which reduces its efficacy in the treatment of hyperlipidaemia. Folate supplementation significantly improves the lipid-lowering and hepatotoxic effects of fenofibrate. This is mainly due to folate-promoting PPARα activation (87). Folate induced a dose-dependent decrease in peroxisome proliferator-activated receptor *γ* (PPARγ), CCAAT/enhancer binding protein α (C/EBPα) gene expression and downstream fatty acid synthetase transcription. Folate supplementation increased adipocyte proliferation and increased the expression of genes involved in OCM, resulting in increased methylation of the C/EPPα promoter during differentiation and decreased expression of PPARγ (88).

Additionally, as AMPK is an endogenous energy sensor that regulates lipid and carbohydrate metabolism, Folate mediates AMPK activation by promoting AMP elevation and activation of its upstream kinase liver kinase B1 (LKB1), Folate supplementation restores AMPK phosphorylation activation and reduces blood glucose and liver cholesterol levels. Folate promotes AMPK-dependent phosphorylation of HMG-CoA reductase, resulting in reduced hepatic cholesterol synthesis during HFD feeding (89). Folate supplementation in high-fructose-fed rats increased the phosphorylation levels of AMPK and LKB1 and inhibited the phosphorylation of ACCs in the liver, then significantly increases hepatic SAMe, inhibits hepatic lipogenesis and thus ameliorates hepatic steatosis (90).

### 4.4 Folate supplementation regulates OCM and DNA methylation

Folate is an important cofactor in methyl metabolism, providing methyl donors to methyltransferases and promoting DNA methylation, affecting DNA mutation rates and genome-wide methylation. Studies have reported that both high and low folate intakes increase *de novo* mutation rates and disrupt genomic DNA methylation in offspring. High folic acid diets significantly increased whole blood folate concentrations, contributing to a 1.8-fold increase in DNA mutation rates. In contrast, the low folic acid diet significantly decreased whole blood folate concentrations, and mice in the low dose group had a 2-fold increase in DNA mutation rates (91). Significant hypermethylation of DNA repair genes in high folic acid diets suggests that excessive folic acid supplementation may impair DNA repair activity by affecting de novo mutation rates through downregulation of DNA repair gene expression. In addition, the inability to generate the BER response to oxidative stress in a folate-deficient environment leads to the accumulation of DNA repair intermediates, inducing DNA strand breaks. Folate deficiency inhibits the upregulation of β-pol expression in response to oxidative stress (92). And excessive folate concentrations lead to DNA base excision to repair DNA damage induced by gene expression. This suggests that folate supplementation should be limited to the desired benefit.

Folate also mediates OCM, regulates DNA methylation and affects hepatic lipid metabolism. Methyl donor supplementation decreased overall DNA methylation levels in the liver of MASLD rats, and methylation levels at specific CpG sites in the promoter regions of genes involved in lipid metabolism, such as LEPR, SREBF2, AGPAT3 and ESR1, were altered by obesity diet and methyl donor supplementation, thereby regulating blood lipids, liver weight and fat content (93). The expression profile of folate-treated hepatocytes showed that folate inhibited lipid deposition by regulating DNA methylation, affecting the transcription and protein levels of genes related to lipid metabolism and the autophagy pathway (94). These studies may provide evidence for the beneficial effects of folate supplementation in the treatment of MASLD.

There is a strong link between maternal lipid metabolism leading to obesity and the development of MASLD in offspring (95). A study from the University of Washington School of Medicine has shown for the first time that genetic variations caused by pre-pregnancy obesity in women can be passed through the bloodstream for more than three generations, leading to obesity and insulin resistance in offspring and increasing the risk of obesity-related diseases in future generations (96). Maternal and post-weaning folate supplementation can significantly modulate global and gene-specific DNA methylation of liver in rat offspring. Maternal one-carbon supplement altered the offspring phospholipid profile programmed by maternal HFD. The maternal HFD elevated sphingomyelin (SM) and dihydrosphingomyelin (DSM) and reduced the proportions of PE and ethyl-linked PE (ePE). The one-carbon supplement normalized SM and DSM, reversed the lower levels of glycerophosphoglycerols (PG) and ePE which were caused by maternal HFD. Besides, one-carbon supplement enhanced PE and increased phosphatidylserine (PS), glycerophosphoinositols (PI), ethyl-linked PS (ePS), and phosphatidic acid (PA), and decreased PC and ethyl-linked PC (ePC) in the offsprings. DNA methylation of PRKCA, DGKH, PLCB1, and DGKI were identified associating with phospholipid metabolism (97). It suggests that maternal HFD disrupts the phospholipid profile of the offspring, leading to exacerbation of hepatic steatosis. Maternal one-carbon supplementation may inhibit hepatic steatosis by regulating DNA methylation modification to prevent phospholipid metabolism disorders.

Mutations in the MTHFR gene lead to a decrease in the activity of the enzyme MTHFR, which affects the decreased production of 5-MTHF, the active form of folate, and affects the normal methylation and synthesis of DNA, as well as leading to an increase in the concentration of tHcy, which is closely associated with the development of HHcy. The encoding of MTHFR appears to be polymorphic, such as gene site C677T, one of the most studied and clinically important variant in exon 4. The C677T variant lowers the affinity of MTHFR and its cofactor, which promotes the thermolability and diminishes the enzyme activity. Comparing with wild genotype (CC), the heterozygote (CT) and mutation homozygote (TT) lead to the decline of enzyme activity by about 34 and 75%. Another common polymorphism is A1298C, which also diminishes the enzyme activity. Homozygous individuals showed 61% of wild-type (98). Therefore, before supplementing with folate, it is necessary to determine the MTHFR gene mutation. If MTHFR mutations are present, tHcy levels also need to be tested. High-dose folic acid supplementation is recommended for MTHFR mutations accompanied by elevated tHcy. Folic acid is not physiologically active and conversion is limited, which can result in an excess buildup of inactive folic acid that may negatively affect the immune system. For people with MTHFR mutations accompanied by elevated blood homozygosity, supplementation with active folate, 5-MTHF, is more desirable. Consumption of folate-rich foods will also support the health of the methylation cycle, thereby reducing the negative effects of MTHFR mutations.

### 4.5 Folate supplementation slows aging

As a central metabolic organ, the liver shows signs of progressive metabolic disorders during aging, such as enhanced TGs accumulation, inhibition of fatty acid oxidation, and impaired lipolysis. In 2024, a striking study reported that mice undergo lipidomic changes during aging, with large lipid accumulations in 10 different tissues, including muscle, kidney, liver, and heart, and in particular, a complex lipid called bis(monoacylglycero)phosphate (BMP) is particularly noteworthy (99). Recently, it has been suggested that dietary folate intake is strongly associated with aging. A cross-sectional study based on the NHANES database from 2007 to 2016, including 10,278 adults, found that higher dietary folate intake was positively associated with elevated serum levels of the longevity factor Klotho. It suggests that higher dietary intake of folate may help to elevate Klotho levels, thereby slowing down aging to some extent (100). The same conclusion was reached in another study that analyzed the folate intake of 18,889 participants from the 2003–2018 NHANES database and found a correlation between higher folate intake and slower biological aging, especially when natural food sources of folate intake were more helpful in slowing down aging (101). Besides, elderly people supplemented with folic acid (400 ug/day) had significantly stronger natural killer cells (NK cells) function and were less susceptible to infections than their peers who were not supplemented with folic acid (102). In addition, folate-mediated one-carbon metabolites affect biological lifespan. It is reported that regulation of the folate cycle represents a shared causal mechanism of longevity and proteoprotection in *Caenorhabditis elegans* (103). In a word, moderate folate supplementation can provide strong support for slowing down aging and reducing the risk of age-related diseases.

### 4.6 Prenatal folate supplementation

There is a strong link between maternal lipid metabolism leading to the development of MASLD in offspring. Folate supplementation effectively improved hepatic lipid accumulation and inflammatory infiltration in male offspring of mothers with a HFD, and maternal folate supplementation reduced the abundance of Desulfobacterota and the ratio of Firmicutes/Bacteroidetes in male offspring (104). These results indicated that folate supplementation during pregnancy could regulate the gut microbiota of male offspring and improve gut barrier integrity, and folate inhibited the expression of the TLR4/NFκB pathway in the liver, thereby ameliorating lipid metabolic disorders and alleviating hepatic steatosis. Folate is a methyl donor required for the synthesis of methionine, a precursor of SAMe, a substrate for methylation. Methyl donor deficiency can lead to hepatic steatosis and a predisposition to metabolic syndrome. Folate deficiency promotes a decrease in methionine synthase activity, impaired mitochondrial fatty acid oxidation, and induced hepatic steatosis.

When developing a nutritional program for pregnancy preparation in dailylife, it has been found that there is a fundamental difference in folate supplementation goals for men and women. Folate has an important role in the development of the neural tube. When folate levels are significantly decreased, the 5-MTHF metabolic pathway is blocked and the SAMe/SAH ratio is down-regulated, which inhibits methyltransferase activity and disrupts DNA methylation homeostasis thus leading to delayed closure of the neural tube, and ultimately to the formation of neural tube defects (NTDs) (105). Therefore, women take folate supplements primarily to reduce the risk of fetal NTDs. Sperm DNA damage is a common cause of male infertility. Folate deficiency increases methylation levels in the Rad54 promoter region, increases *γ*-H_2_AX and induces DNA damage (106). Therefore men take folate supplements to improve sperm DNA fragmentation rates. Although no significant gender differences have been found to exist in liver diseases. However, one study found a sex difference in folate dietary treatment, with male mice being more sensitive to high folate dietary treatment compared to female mice. This difference may be related to sex differences in germline stem cell proliferation, as male mice continue to produce new sperm (91).

### 4.7 Opposite opinion on folate supplementation

It is now widely recognized that folate supplementation can mitigate the development and evolution of metabolic diseases. However, other reports have come to the opposite conclusion. A review of randomized controlled trials published in the PubMed, EMBASE, Web of Science and Cochrane Library databases concluded that folate supplementation had no effect on blood pressure and lipids in patients with metabolic diseases (95). This suggests that folate supplementation should be carefully considered in various diseases, especially in patients with metabolic diseases. In some clinical situations, folate may have adverse effects. Folate administration was associated with increased hepatic inflammation and apoptosis and exacerbated fibrosis in CCL4-treated rats (107). Therefore, folate supplementation has certain limitations in the treatment of patients with chronic liver diseases.

Although prenatal folate supplementation is recommended in many countries, a number of studies have found some drawbacks to folate supplementation. Prenatal folate supplementation in female Sprague–Dawley rats on a HFD significantly increased serum and hepatic TGs levels in their male offspring. Prenatal folate supplementation is necessary. However, excessive folate supplementation leads to mild dyslipidaemia diabetes in male offspring, and increasing the dose of folate may lead to further fat accumulation in the liver (108). Although prenatal folate supplementation is recommended in many countries, a number of studies have found some drawbacks to folate supplementation. Prenatal folate supplementation in female Sprague–Dawley rats on a HFD significantly increased serum and hepatic TGs levels in their male offspring. Maternal prenatal folate supplementation induced aberrant DNA methylation of adipose triglyceride lipase (ATGL) in the liver and LPL in white adipose tissue, resulting in a significant decrease in the expression of ATGL and LPL, exacerbating the adverse effects of HFD on lipid metabolism in their offspring (109). Maternal high-fat, high-sugar diet alters insulin sensitivity and hepatic *de novo* lipogenesis in offspring, while prenatal folate supplementation induces IR (110). Therefore, in addition to prenatal folate supplementation, dietary modifications may need to be considered to ensure lipid metabolic homeostasis in the offspring.

## 5 Anti-MASLD/MASH drugs

**Figure 5:**
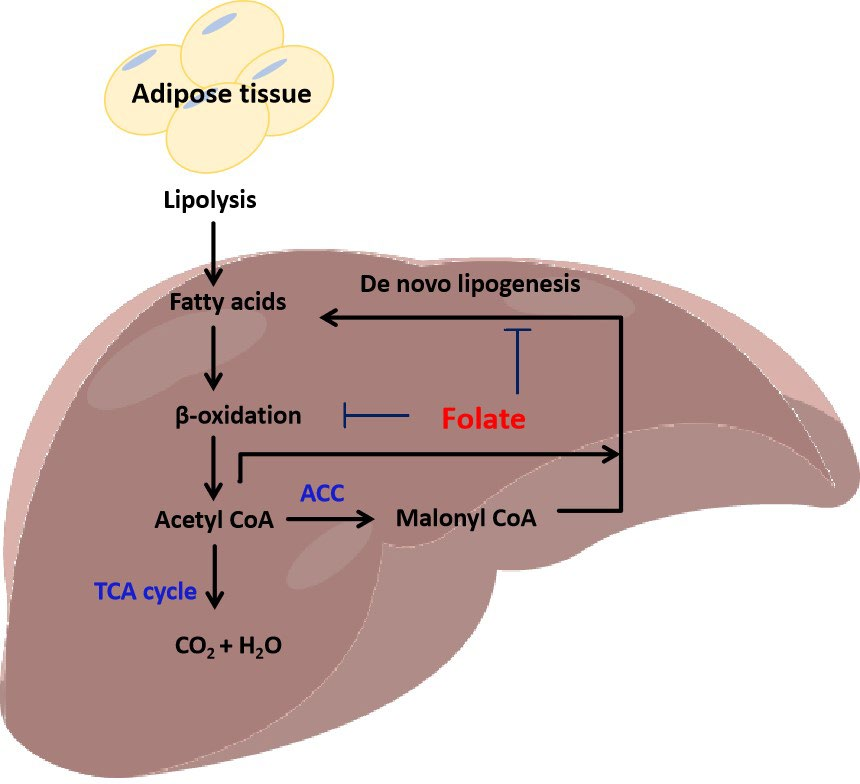
Folate inhibits fatty acid β-oxidation and *de novo* lipogenesis in the liver.

Oxidative stress, inflammation, obesity, T2DM, IR, gut microbiology, and epigenetic regulation are all thought to play roles in the onset and progression of MASLD and MASH. There are three main kinds of drugs currently used against MASLD and MASH: metabolic regulators including drugs that regulate glucose, lipid, and bile acid metabolism, antifibrotic drugs, and anti-inflammatory drugs (Figure 5).

Metabolic regulators target abnormalities in glucose, lipid, and bile acid metabolism in order to restore normal liver function. Glucagon-like peptide-1 receptor (GLP1R) agonists, fibroblast growth factor 21 (FGF21), and sodium-glucose cotransporter protein 2 (SGLT2) inhibitors are used to improve insulin sensitivity, regulating glucose metabolism and reducing hepatic fat accumulation. Thyroid hormone receptor β (THRβ) agonists, and ACC inhibitors are used to reduce hepatic fat synthesis and promote fatty acid oxidation, thereby reducing hepatic fat content. PPAR agonists and farnesol X receptor (FXR) agonists are used to improve lipid homeostasis and inhibit inflammatory responses by regulating bile acid synthesis, secretion and metabolism. Anti-fibrotic drugs block or reverse the process of hepatic fibrosis, such as Galatin-3 antagonists, CCR2/CCR5 antagonists and PPAR agonists, which slow or reverse the progression of fibrosis by inhibiting HSCs activation, decreasing collagen deposition and inhibiting inflammatory signaling. Besides, anti-inflammatory drugs including CCR2/CCR5 antagonists, FGF19 and FGF21 mimetics, and TNFα antagonists, which alleviate the inflammatory by inhibiting the infiltration of inflammatory cells, modulating the immune response, and reducing inflammatory mediators (111, 112). In addition, metformin is often used in the treatment of T2DM, also improves MASLD by reducing hepatic gluconeogenesis and TGs production. Anti-oxidants such as vitamin E is capable of repairing oxidizing radicals and prevent lipid peroxidation (113). Gut microbes play a role in the development of MASLD and MASH. Gut microbe-modulating drugs, such as prebiotics, probiotics, and specific antibiotics, may positively affect MASLD and MASH by altering gut microbial composition.

**Figure 6:**
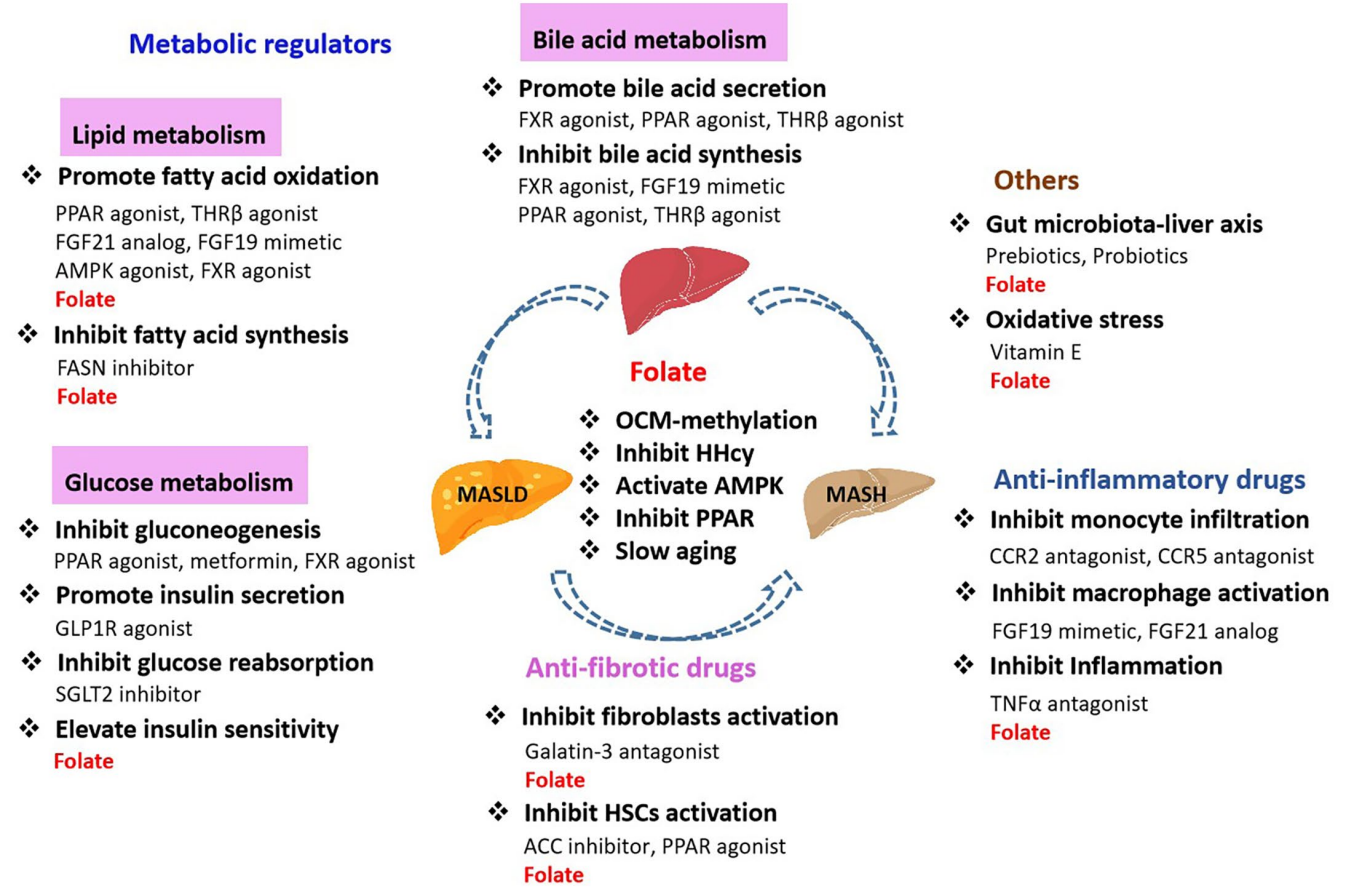
Anti-MASLD/MASH drugs.

Folate regulates hepatic lipid metabolism by modulating a variety of mechanisms in MASLD, such as inhibiting fatty acid synthesis, promoting fatty acid oxidation, mediating OCM affects DNA methylation, regulating mitochondrial function and influencing hepatic metabolism (7). Folate regulates PPAR and AMPK signaling pathways, which in turn affect HSCs activation, and have a role in the development of MASLD to MASH. HHcy correlated with hepatic inflammation and fibrosis in MASH. Elevated Hcy induced and exacerbated MASH. HHcy plays a key role in the pathogenesis of MASH via Syntaxin 17 (Stx17) homocysteinylation. Dietary vitamin B12 and folate, which promotes enzymatic conversion of Hcy to methionine, decreased HHcy, and restored Stx17 and autophagy, stimulated β-oxidation of fatty acids, and improved hepatic histology in mice with pre-established MASH (114), suggesting that vitamin B12 and folate could have therapeutic potential for the prevention or treatment of MASH. These suggest that folate has the potential to serve as a potential therapeutic agent for the development of MASLD and the onset of MASH. Anti-MASLD/MASH drugs as shown in Figure 6.

## 6 Concluding remarks

According to the mechanism of MASLD, drugs for MASLD are mainly targets the gut-liver axis to treat MASH by regulating the interaction between the intestine and the liver, improving metabolism, adjusting metabolic abnormalities associated with MASH pathogenesis, and anti-inflammatory drugs that inhibit the chronic inflammation that accompanies MASH. Rezdiffra, which is the first and the only one approved by the FDA for the treatment of MASH, is a partial agonist of THRβ that acts on thyroid hormone receptors in the liver. Though this review summarizes the role of folate in regulating hepatic lipid metabolism in the onset and development of MASLD, through different mechanisms, such as regulation of hepatic lipid metabolism, signaling pathways, OCM, aging, etc. Folate is still not a treatment drug for MASH, it should only be used as an adjunctive therapy. For patients with MASH, proper supplementation of folate can prevent the symptoms of anemia that may occur. Besides, folate can help repairing and regenerating liver cells when liver is impaired. Both studies emphasized the importance of natural food sources of folate, rather than folic acid supplements (100, 101), and that higher natural food folate intake, and lower folic acid supplements, are what help to slow down aging. The reason why natural folate is significantly more effective than folic acid supplements may be due to the fact that folic acid supplements are metabolized to produce products such as tetrahydrofolate, which can have adverse health effects. Keeping a balanced diet and consuming folate from everyday foods may be a better choice for boosting Klotho levels and promoting normal lipid metabolism. Folate-rich foods such as green leafy vegetables, citrus fruits, legumes, nuts and whole grains should be a daily choice in our diet. As MTHFR gene mutation leads to low levels of folate and vitamin B, and high levels of tHcy. The use of blood MTHFR genotype testing has the potential to help identify the cause of the disease by clarifying whether MTHFR mutations are present and affecting the way the body processes folate, which can help in the treatment of the disease. Some daily habits can also help to minimize the effects of the MTHFR mutation.

Despite the known beneficial effects of folate, there are still some limitations to folate supplementation, and appropriate clinical trials are necessary to determine the optimal dosage of folate supplementation for specific populations to minimize adverse effects. In general, folate regulates hepatic lipid metabolism through a variety of mechanisms, playing a role in delaying liver metabolism, inhibiting fatty acid synthesis, promoting fat oxidation, and reducing oxidative stress and inflammation. The multifaceted effects suggest that folate supplementation may have a potential impact on the treatment of MASLD.
